# Post-marketing surveillance study on the effectiveness and safety of molnupiravir in high-risk COVID-19 outpatients: a prospective case series study

**DOI:** 10.1007/s43440-025-00729-2

**Published:** 2025-04-25

**Authors:** Renato Ferreira-da-silva, Lurdes Silva, Cristina Costa-Santos, Manuela Morato, Jorge Junqueira Polónia, Inês Ribeiro-Vaz, Manuela Pinto, Marta Pereira, Inês Marques Figueira, Sofia Baptista, Helena Farinha, Fátima Falcão, Ana Mirco, Liliana Calixto, Madalena Melo, ESOA-19 Research Team

**Affiliations:** 1 Porto Pharmacovigilance Centre, Faculty of Medicine of the University of Porto, Porto, Portugal; 2 Department of Community Medicine, Information and Health Decision Sciences (MEDCIDS), Faculty of Medicine of the University of Porto, Porto, Portugal; 3 RISE-Health, Porto, Portugal; 4 Laboratory of Pharmacology, Department of Drug Sciences, Faculty of Pharmacy of the University of Porto, Porto, Portugal; 5 LAQV@REQUIMTE, Faculty of Pharmacy of the University of Porto, Porto, Portugal; 6 Department of Medicine, Faculty of Medicine of the University of Porto, Porto, Portugal; 7 Local Health Unit of São João, EPE, Porto, Portugal; 8 Local Health Unit of Santo António, EPE, Porto, Portugal; 9 Local Health Unit of Vila Nova de Gaia/Espinho, EPE, Vila Nova de Gaia, Portugal; 10 Family Health Unit - Homem do Leme (ACES Porto Ocidental), Porto, Portugal; 11 Local Health Unit of Lisboa Ocidental, EPE, Lisboa, Portugal; 12 Divino Espírito Santo Hospital - Ponta Delgada, EPE, Azores, Portugal

**Keywords:** SARS-CoV-2, COVID-19 drug treatment, Antiviral agents, Molnupiravir, Post-marketing surveillance, Adverse drug events

## Abstract

**Background:**

Molnupiravir, approved for treating mild to moderate COVID-19 in adults, aims to reduce hospitalisation and mortality rates. Although it was withdrawn from the market after the present study was conducted, understanding its long-term effects remains pertinent. We aimed to assess the real-world effectiveness and safety of molnupiravir in high-risk COVID-19 outpatients.

**Methods:**

This prospective, multicenter, noninterventional, postmarketing cohort study enrolled high-risk COVID-19 outpatients with mild to moderate COVID-19, eligible under national prescribing criteria, who initiated molnupiravir within five days of symptom onset and were ineligible for first-line antiviral therapy. Patients were consecutively enrolled from eight Portuguese study sites and monitored for three months. Effectiveness was assessed by all-cause mortality and hospitalisation through day 29. Safety was evaluated by the incidence, severity, and causality of adverse events (AE), coded using MedDRA terminology and assessed via the WHO-UMC system. Data were collected through structured patient questionnaires and electronic health records. Statistical analysis was descriptive; proportions were reported with 95% confidence intervals (CI), and comparisons between groups were performed using appropriate statistical tests.

**Results:**

By day 29 post-treatment initiation, no deaths were reported (n = 0; 0%; 95%CI = [0,26]), and all patients were either at home or institutionalised, with favourable outcomes. Out of the 12 patients enrolled, eight (67%; 95%CI = [35,90]) reported at least one AE, with the median time to the first AE being five days (range 5–7 days). Half of the patients (n = 6; 95%CI = [21,79]) reported AE deemed possibly or probably related to molnupiravir, involving nausea (25%), dizziness (17%), bitter taste (17%), and headache (17%). These AE were more commonly observed in older individuals and those overweight, indicating a potential influence of these factors on AE occurrence.

**Conclusions:**

Molnupiravir appears to show good safety and effectiveness, offering an alternative for high-risk COVID-19 outpatients ineligible for first-line therapy. Despite its market withdrawal, ongoing research into its long-term effects is crucial to potentially repurpose it for other viral infections.

**Supplementary information:**

The online version contains supplementary material available at 10.1007/s43440-025-00729-2.

## Key points

• Our study suggests that molnupiravir is safe and effective, offering an alternative treatment option for high-risk COVID-19 outpatients who are ineligible for first-line therapy.

• Molnupiravir was associated with no instances of all-cause mortality or hospitalisation among high-risk COVID-19 patients, highlighting its effectiveness in preventing severe disease progression.

• The most frequently reported AE were nausea, dizziness, bitter taste, and headache, with nausea appearing as early as one day post-treatment, persisting for up to three months, and being notably serious in one-fourth of these instances.

• AE with attributed causality were more frequently described in older or overweight individuals, suggesting these factors might influence the occurrence of AE with molnupiravir.

• Despite recent molnupiravir’s market withdrawal, continued research on its long-term effects is crucial to repurposing it for other viral infections and advancing medical knowledge based on the experiences of treated patients.

**Supplementary information:**

The online version contains supplementary material available at 10.1007/s43440-025-00729-2.

## Introduction

In the wake of the COVID-19 pandemic, the pressing demand for effective and safe therapeutic interventions led to a significant shift in the approach to treating viral infections. The initial strategy, centred around repurposing existing drugs, produced mixed results in terms of efficacy. This inconsistency highlighted the critical need for novel, direct-acting antiviral agents specifically formulated to combat SARS-CoV-2, the novel coronavirus causing COVID-19 [1]. Administering these drugs early in the community setting could potentially mitigate symptom severity, expedite recovery, and decrease viral shedding, thereby reducing hospital admissions and lessening healthcare burden. Among these new therapeutic options, molnupiravir emerged as a noteworthy candidate. Initially developed for treating influenza [2], molnupiravir was repurposed and assessed for its efficacy against COVID-19 [3], demonstrating the adaptive and dynamic nature of the medical response to the pandemic.

Molnupiravir operates through a novel mechanism as a ribonucleoside analogue, targeting the RNA-dependent RNA polymerase (RdRp) of SARS-CoV-2, essential for the replication and transcription of its RNA. This drug is metabolised into β-d–N4-hydroxycytidine triphosphate in cells, where it competes with endogenous nucleotides and gets incorporated into the viral RNA [4]. Once incorporated, molnupiravir induces a state of ‘viral error catastrophe’ by causing an accumulation of mutations within the viral genome. This process effectively hampers the virus’ ability to replicate accurately [5]. Its efficacy in reducing viral replication not only aids in managing mild to moderate COVID-19 cases but also decreases viral shedding, which is significant for reducing transmission rates and easing healthcare burdens [5]. Molnupiravir demonstrated effectiveness against SARS-CoV-2 in various animal studies [6, 7] and has been found to be safe and well-tolerated in human subjects at a dosage of 800 mg twice daily, as evidenced in phase 1 trials [8, 9], and later confirmed in phase 2 and 3 trials conducted in outpatient settings [3, 10, 11].

In the MOVe-OUT trial, a comprehensive phase 3 study funded by the industry and conducted with a placebo-controlled design, unvaccinated, non-hospitalized high-risk COVID-19 patients were primarily evaluated. The trial’s modified intention-to-treat interim analysis revealed a consistent reduction in hospitalisation or death rates in the molnupiravir group compared to placebo. An early report showed that molnupiravir cut the risk of hospitalisation and death to 50% (7.3% in molnupiravir group vs. 14.1% in placebo group) [12] in patients who had mild-to-moderate disease, but a final analysis of the trial, before the FDA authorisation, showed that reduction to be 30% (6.8% in molnupiravir group vs. 9.7% in placebo group) [11].

As such, molnupiravir became available under conditional authorisation, being predominantly used as a second-line therapy, prescribed mainly to those patients ineligible for first-line treatment with ritonavir/nirmatrelvir [14]. Consequently, its usage was limited to a relatively small patient segment, resulting in a notably lower number of individuals receiving this treatment during its period of authorised use [15]. In February 2023, the European Medicines Agency (EMA) declined the authorisation for the market introduction of molnupiravir [13], citing the latest data (30%) that failed to conclusively demonstrate the drug’s ability to reduce the risk of hospitalisation or death or to shorten the duration or recovery time of the disease in adults with COVID-19 at risk of progressing to serious illness.

The scenario with molnupiravir, initially developed for influenza and later repurposed for COVID-19, suggests its potential future use in other viral infections [16]. Despite the current scarcity of observational evidence on molnupiravir’s real-world effectiveness and safety in treating COVID-19 [15, 17–19], acquiring this knowledge could be essential for its potential use in other clinical conditions in the future. Moreover, understanding the long-term effects of molnupiravir is crucial for the comprehensive care of patients who have been treated with this medication. Gaining this knowledge is crucial not only for current treatment outcomes but also for preparing for future clinical implications for those exposed to these drugs, ensuring their experiences and outcomes are integral to ongoing medical research and practice.

In this study, we aimed to explore the real-world effectiveness and safety of molnupiravir administered to COVID-19 outpatients who were at high risk of developing serious disease. The findings offer crucial insights to guide future political, regulatory, and clinical decision-making, both for COVID-19 and potentially other clinical conditions.

## Methods

### Study design & setting

This is a phase IV, open, prospective, multicenter, noninterventional, postmarketing cohort study. The study’s design involved cohort event monitoring for three months following the initiation of treatment with molnupiravir. The study was conducted in Portugal and incorporated five hospital centres in Porto and Lisbon, translating into seven study sites alongside one hospital centre in the Azores. The study centres were primarily hospital-based but also extended to primary healthcare facilities where the medication was dispensed. These sites were selected based on Norm nr 005/2022 of May 28, 2022, issued by the General Directorate of Health (*Direção Geral da Saúde*, DGS) of Portugal. This directive informed the inclusion of both primary healthcare centres and hospital settings, specifically through outpatient pharmacy services, in the dispensation of oral antiviral drugs for COVID-19.

The study protocol was initially approved by the Ethics Committee for Health of the São João University Hospital Centre (CES nr. 202/2022) and subsequently by the ethics committees of the other participating centres. It was conducted by the principles outlined in the Declaration of Helsinki and the Oviedo Convention, ensuring strict adherence to ethical standards and the protection of personal data. All patients provided informed, voluntary, explicit, and written consent to participate in the study. No one received compensation or was offered any incentive for participating in this study.

The study is registered on clinicaltrials.gov (NCT05894603) and in the Catalogue of RWD Studies (EUPAS48186), having received the study seal from the European Network of Centres for Pharmacoepidemiology and Pharmacovigilance (ENCePP), which signifies that it adheres to ENCePP’s core principles of scientific independence, transparency, and robust methodologies.

### Study population

The study population comprised patients who had received a medical prescription for molnupiravir at the eligible study centres. For inclusion, participants or their representatives were required to fulfil specific criteria as outlined by Norm nr 005/2022 of the DGS. These criteria were comprehensive, including (i) patients aged 18 years or older at the index date, (ii) ability to understand the Portuguese language, (iii) availability for follow-up, (iv) diagnosis of mild to moderate COVID-19 without the need for new oxygen therapy, (v) experiencing symptoms for less than five days at index date, and (vi) either suffering from serious immunodepression regardless of vaccination status or being at risk for a serious disease without COVID-19 infection or vaccination in the preceding six months. The exclusion criteria included (i) individuals currently participating in any phase I–IV clinical trials and (ii) those with a life expectancy of less than one month. These criteria for patient selection for therapy initiation followed the established standard care, requiring no additional intervention from the research team.

For the present study, patients were identified based on electronic prescriptions by Pharmacy service from September 2022 to September 2023, with pharmacists tasked with patient recruitment within 72 hours after dispensing the medication at the outpatient pharmacy during the recruitment period from September 2022 to September 2023. After the patient expressed informed consent and met the inclusion criteria, the patient was immediately recruited and included in the study database. This timeframe was essential to ensure that the health data collected before starting treatment was clear and not affected by any changes due to the therapy’s initiation.

The index date was established as the date of the first prescription of molnupiravir. Correspondingly, the index treatment was defined as the first day when treatment with molnupiravir commenced. Subjects could voluntarily withdraw from the study for any reason at any time if they state an intention to withdraw or become lost to follow-up contacts for any other reason.

This study is part of the larger ESOA-19 study, which includes nirmatrelvir/ritonavir monitoring. As such, this molnupiravir-focused study did not have a distinct sample size calculation due to the unpredictable demand for molnupiravir as a second-line treatment. Therefore, we adopted a consecutive sampling method, systematically including every patient prescribed molnupiravir at the study centres. The local investigator supervised the adherence to the study protocol.

### Data sources

Data was collected directly from patients using structured forms developed and pre-tested by the research team, informed by existing literature and the clinical expertise of the team members. This process involved administering a questionnaire to patients through periodic telephone calls, ensuring comprehensive recording of their responses. Local investigators were instructed to gather information from electronic health records (EHR) for variables such as comorbidities and co-medication. Additionally, information related to medication exposure, including dosages, durations, and any modifications to the prescribed regimen, was also meticulously extracted from the EHR. This dual approach of direct patient questioning and EHR data extraction ensured a thorough and accurate compilation of data, crucial for the robustness of the study’s findings.

### Drug use & covariates

**Fig. 1:**
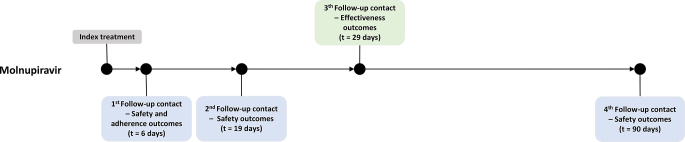
Timeline for baseline and follow-up assessments

The questionnaire was structured into three sections. The initial section collected sociodemographic data, such as year of birth, gender, height, weight, professional activity, and information on smoking habits and current tobacco use. The subsequent section was dedicated to gathering clinical data, which included details of exposure to molnupiravir, the patient’s comprehensive medical history, and any changes in health status observed over the past two weeks before index treatment, specifically noting any signs or symptoms indicative of COVID-19. The final section was designed for ongoing patient monitoring, aiming to evaluate the safety and effectiveness of the treatment with molnupiravir. The safety outcome was described as the incidence of AE, with a particular focus on AE of special interest that emerged during or after the treatment period, as serious AE and AE leading to treatment discontinuation, and coded according to the MedDRA terminology. Incidence data included all patients who received at least one molnupiravir dose. The occurrence of AE was inquired about through open, unsolicited questions without directly mentioning any specific connection to the medication. For each reported AE, the date of onset, outcome, duration of symptoms (if recovered), and severity/impact of the symptoms (including medical assistance and hospitalisation) were inquired. All adverse events considered to have a probable or possible causal relationship with molnupiravir were formally reported to the Portuguese Pharmacovigilance System, of which the authors are active members. The effectiveness outcome was described as the incidence of hospitalisation for any cause (defined as ≥ 24 hours of acute care in a hospital or any similar facility) or death for any cause through day 29 (Fig. 1). Additionally, some questions were collected regarding patient adherence to molnupiravir treatment.

Follow-up contacts were strategically timed to assess both short-term and long-term outcomes (Fig. 1). Anticipating that most AE would manifest within 5 to 14 days after beginning treatment [20], the first safety follow-up was scheduled for the 6^th^ day post-treatment onset. This timing was crucial for promptly capturing initial reactions. Subsequently, a second follow-up on the 19^th^ day post-treatment onset with molnupiravir aimed to identify any later-occurring AE and to gather additional details about the recovery from previously reported AE. Additionally, a third safety follow-up was conducted 3 months after the commencement of treatment for both cohorts, providing insights into the long-term safety profile of the treatment. Treatment adherence outcomes were evaluated on the 6^th^ day after treatment onset. The follow-up contact periods were considered valid until a maximum of five days after the expected date of contact for assessing safety and adherence outcomes and a maximum of two months after the expected date for assessing effectiveness outcomes.

The study followed the STROBE (Strengthening the Reporting of Observational Studies in Epidemiology) guidelines [21] for reporting. A STROBE checklist can be found in the supplementary material (Table S1.) [22]. Additionally, the study adheres to the methodological standards outlined in the ENCePP Guide on Methodological Standards in Pharmacoepidemiology.

### Statistical analysis

All AE were evaluated for causality through an assessment grounded in global introspection. This evaluation considered factors such as pharmacological plausibility, severity and intensity, causal nexus, predisposing factors, the impact of treatment discontinuation or completion, concomitant medication, symptoms of underlying diseases, and temporality. Only AE classified as probable or possible on the WHO scale [23] were considered to have causality with molnupiravir. Causality assessment was conducted by a team of pharmacovigilance professionals, including physicians and pharmacists, in accordance with the global introspection method used by the Portuguese Pharmacovigilance System.

A descriptive analysis was conducted on sociodemographic (age over 60 years and gender) and clinical (smoking habits, drug allergies, and history of SARS-CoV-2 infection) variables. The statistical methods applied included the Mann-Whitney U test, particularly focused on age and BMI.

The study estimated the frequencies of each AE with respective 95% confidence intervals (CI). Median AE duration and median time until the onset of the first AE, along with their minimum and maximum values, were calculated for these events. Sub-analyses focused on AE linked to molnupiravir exposure that were classified as probable or possible. Comparisons of sociodemographic and clinical variables between patients with and without at least one probable or possible AE were conducted using the Mann-Whitney U test, or Fisher’s exact test, as appropriate. In cases involving variables such as a history of renal failure or transplant, clinical record data was preferred for its higher reliability compared to patient self-reports. When patients were unable to provide specific details, data were obtained from the EHR to enhance the quality and reliability of the information collected.

For the categorisation of AE, those that occurred on the same day as the initiation of the index treatment were recorded with a time-to-onset of zero days. Similarly, AE that started and ended within the same day was also noted as having a zero-day duration (i.e., any period less than 24 hours).

All statistical tests’ significance level was 5% (p < 0.05). These analyses were performed using SPSS Software (Statistical Package for the Social Sciences), version 29.0.

## Results

### Patient baseline characteristics

The cohort comprised 12 outpatients with mild to moderate COVID-19 (Table 1), with the majority being over the age of 60 years (n = 8, 67%). Females constituted 58% (n = 7) of the study population. Regarding smoking habits, 58% (n = 7) were ex-smokers, while current smokers and non-smokers accounted for 17% (n = 3) and 25% (n = 2), respectively. Drug allergies were present in 58% (n = 7) of participants. A significant proportion of the cohort reported current severe SARS-CoV-2 infection or COVID-19 based on their symptoms (n = 8, 67%), although they were classified as mild to moderate cases in the outpatient setting. Additionally, half of the patients (n = 6) had a history of prior SARS-CoV-2 infection or COVID-19.

**Table 1:** Sociodemographic and clinical characteristics of high-risk COVID-19 outpatients treated with molnupiravir in a prospective post-marketing case series conducted across eight public healthcare centres in Portugal (Divino Espírito Santo Hospital - Ponta Delgada, Local Health Unit of Lisboa Ocidental, Local Health Unit of Vila Nova de Gaia/Espinho, Local Health Unit of São João, Local Health Unit of Santo António, Family Health Unit - Homem do Leme (ACES Porto Ocidental)) from September 2022 to September 2023 (N = 12). Data are presented as number (percentage). Clinical information was collected via structured patient interviews and validated through electronic health records

Description of the population (n = 12)	n (%)
Over 60 years	8 (67)
Female gender	7 (58)
Smoking habits	
Former smoker	7 (58)
Smokers	3 (17)
Non-smoker	2 (25)
History of drug allergies	7 (58)
Current serious SARS-CoV-2 infection or COVID-19	8 (67)
Previous SARS-CoV-2 infection or COVID-19	6 (50)

### Effectiveness outcome

On the 29^th^ day following the initiation of treatment, there were no reported deaths from any cause (n = 0; 0%; 95%CI = [0, 26]), and all patients were at home or institutionalised with favourable outcomes. Also, three months into the monitoring period, all patients were alive and could be contacted for the final safety follow-up. All patients remained alive and were successfully contacted for the final safety follow-up at three months.

### Safety outcome

Out of the 12 patients enrolled, eight (67%; 95%CI = [35, 90]) reported at least one AE. The median time to onset of the first AE was 5 days (range 5–7 days). Focusing only on AE classified as possible or probable compared to molnupiravir treatment, 50% (n = 6; 95% CI 21–79) of the cases reported at least one such AE, with the median of 2.5 AE per patient (range 1–5 AE). The median time to onset of the first possible or probable AE was 5 days (range 5–6 days).

Table 2 shows the AE experienced by participants during the monitoring period for all levels of causality. Nausea was the most frequently reported AE, occurring in one-fourth of patients, with a median time to onset of 1 day (range 0–12 days) and a median duration of 90 days (range 8–90 days), where one case persisted until the end of the monitoring period. Dizziness was also reported by 17% (n = 2) of patients, manifesting after a median of 2.5 days (range 1–4 days) with a median duration of 32.5 days (range 0–65 days). Other AE, including bitter taste and headache, were observed in 17% of the patients, with median durations of 2.5 and 46 days, respectively. It is also noteworthy that the majority of AE were non-serious and resolved without long-term consequences. Notably, serious AE were reported in the context of nausea (33% of cases considered serious, one of the three). Although only one case of each AE of vomiting, anaemia, increased heart rate and kidney dysfunction were reported, all of these were considered serious. The median time to onset for these serious AE ranged from 1 to 45 days, while their duration varied, with some events, such as vomiting and anaemia, persisting until the end of the study period. All individuals who experienced serious AE, such as kidney dysfunction, reported persistence of these events for the full 90-day duration of the study.

**Table 2:** Frequency, time to onset, duration, and seriousness of all adverse events (AE), regardless of causality, reported during a prospective post-marketing case series of high-risk COVID-19 outpatients treated with molnupiravir across eight public healthcare centres in Portugal (Divino Espírito Santo Hospital - Ponta Delgada, Local Health Unit of Lisboa Ocidental, Local Health Unit of Vila Nova de Gaia/Espinho, Local Health Unit of São João, Local Health Unit of Santo António, Family Health Unit - Homem do Leme (ACES Porto Ocidental)) from September 2022 to September 2023 (N = 12). AE were coded using MedDRA preferred terms. Data are presented as number (percentage), 95% confidence interval (CI), median time to AE onset (days), median AE duration (days), and percentage of cases requiring medical assistance. AE with unresolved outcomes at the 3-month follow-up were considered to have a duration of 90 days. CI: confidence interval; AE: adverse event; MedDRA: Medical Dictionary for Regulatory Activities

EA MedDRA description	n (%)	95% CI (%)	Time to AE onset Median (min-max)	AE Duration (days) Median (min-max)	Serious*** %
Nausea	3 (25)	5–57	1 (0–12)	90 (8–90)*	33
Dizziness	2 (17)	2–48	2,5 (1–4)	32,5 (0–65)	0
Taste bitter	2 (17)	2–48	4 (3–5)	2,5 (2–3)	0
Cough	2 (17)	2–48	4,5 (0–9)	49.5 (9–90)**	0
Headache	2 (17)	2–48	6.5 (1–12)	46 (2–90)**	0
Vomiting	1 (8)	0–38	1	65	100
Heart rate increased	1 (8)	0–38	28	28	100
Anaemia	1 (8)	0–38	45	90*	100
Kidney dysfunction	1 (8)	0–38	45	90*	100
Asthenia	1 (8)	0–38	0	90*	0
Cold sweat	1 (8)	0–38	0	11	0
Constipation	1 (8)	0–38	1	4	0
Dysphagia	1 (8)	0–38	1	4	0
Nasal stuffiness	1 (8)	0–38	1	2	0
Tiredness	1 (8)	0–38	1	13	0
Increased appetite	1 (8)	0–38	4	10	0
Dysgeusia	1 (8)	0–38	5	2	0
Arrhythmia	1 (8)	0–38	5	0	0
Anosmia	1 (8)	0–38	5	2	0
Antibiotic-associated diarrhoea	1 (8)	0–38	9	1	0
Stomach pain	1 (8)	0–38	12	90**	0
Muscular pain	1 (8)	0–38	12	90**	0
Swollen tonsils	1 (8)	0–38	13	6	0
Nasal bleeding	1 (8)	0–38	80	90**	0

* Two cases remain without recovery at the date of monitoring conclusion (3 months); thus, a duration of 3 months has been assumed for each

** One case remains without recovery at the date of monitoring conclusion (3 months); therefore, a duration of 3 months has been assumed

*** Medical assistance required

*The adverse event “cough” includes both dry cough and cough with expectoration*

Considering the patients reporting probable or possible AE associated with molnupiravir (Table 3), nausea was experienced by one-third of patients, with durations extending up to 90 days. Dizziness and bitter taste were reported by 17% (n = 2) of patients, with dizziness resolving within a median of 32.5 days and bitter taste within 2.5 days. Headache was also reported by 17% (n = 2) of the patients, persisting for a median of 46 days. While the majority of AE were non-serious, vomiting, reported by 8% (n = 1) of the cohort, was considered serious and lasted for a median duration of 65 days. Cough, experienced by 8% (n = 1), and muscular pain, also reported by 8% (n = 1), both persisted for the full duration of the study, marked at 90 days. Other AE, such as constipation, dysphagia, nasal stuffiness, tiredness, and stomach pain, were each reported by 8% (n = 1) of patients, with individual durations ranging from 2 to 90 days, again underscoring the variable nature of these events.

**Table 3:** Frequency, time to onset, duration, and seriousness of adverse events (AE) with probable or possible causality attributed to molnupiravir, reported in a prospective post-marketing case series of high-risk COVID-19 outpatients treated across eight public healthcare centres in Portugal (Divino Espírito Santo Hospital - Ponta Delgada, Local Health Unit of Lisboa Ocidental, Local Health Unit of Vila Nova de Gaia/Espinho, Local Health Unit of São João, Local Health Unit of Santo António, Family Health Unit - Homem do Leme (ACES Porto Ocidental)) from September 2022 to September 2023 (N = 12). AE were classified using WHO-UMC global introspection criteria and coded with MedDRA preferred terms. Data include number (percentage), 95% confidence interval (CI), median time to onset and AE duration (days), and proportion of serious cases requiring medical assistance. AE with unresolved outcomes at 3-month follow-up were assumed to have a duration of 90 days. AE: adverse event; CI: confidence interval; MedDRA: Medical Dictionary for Regulatory Activities

EA MedDRA description	n (%)	95% CI (%)	Time to AE onset Median (min-max)	AE Duration (days) Median (min-max)	Serious*** %
Nausea	3 (25)	5–57	1 (0–12)	90 (8–90)**	33
Dizziness	2 (17)	2–48	2.5 (1–4)	32.5 (0–65)	0
Taste bitter	2 (17)	2–48	4 (3–5)	2,5 (2–3)	0
Headache	2 (17)	2–48	6.5 (1–12)	46 (2–90)*	0
Vomiting	1 (8)	0–38	1	65	100
Constipation	1 (8)	0–38	1	4	0
Dysphagia	1 (8)	0–38	1	4	0
Nasal stuffiness	1 (8)	0–38	1	2	0
Tiredness	1 (8)	0–38	1	13	0
Cough	1 (8)	0–38	9	90*	0
Stomach pain	1 (8)	0–38	12	90*	0
Muscular pain	1 (8)	0–38	12	90*	0

* One case remains without recovery at the date of monitoring conclusion (3 months); therefore, a duration of 3 months has been assumed

** Two cases remain without recovery at the date of monitoring conclusion (3 months); thus, a duration of 3 months has been assumed for each

*** Medical assistance required

*The adverse event “cough” includes both dry cough and cough with expectoration*

When comparing patients who reported at least one probable or possible AE with those who did not report any such AE (Table 4), we observed some differences, although they were not statistically significant. Among those with AE, 68% (n = 4) were female, compared to 50% (n = 3) among those without AE. Additionally, there was a non-significant difference in age; the median age for patients with AEs was 68 years (IQR 62–79), whereas it was 42 years (IQR 24–66) for patients without AE.

**Table 4:** Comparison of demographic and clinical variables between patients with and without at least one probable or possible adverse event (AE) attributed to molnupiravir in a prospective post-marketing case series conducted across eight public healthcare centres in Portugal (Divino Espírito Santo Hospital - Ponta Delgada, Local Health Unit of Lisboa Ocidental, Local Health Unit of Vila Nova de Gaia/Espinho, Local Health Unit of São João, Local Health Unit of Santo António, Family Health Unit - Homem do Leme (ACES Porto Ocidental)) from September 2022 to September 2023 (N = 12). Data are presented as number (percentage) for categorical variables and median (1st–3rd quartile) for continuous variables. AE: adverse event; BMI: body mass index

	Probable or possible AE		p
	Without any	With at least one	
Female gender, *n (%)*	3 (50)	4 (68)	1.000
Age, *median (1*^*st*^*-3*^*rd*^* quartile)*	42 (24–66)	68 (62–79)	0.077
BMI, *median (1*^*st*^*-3*^*rd*^* quartile)*	24 (20–29)	26 (22–31)	0.522
Smoking habits, *n (%)*			0.545
Former smoker	3 (50)	4 (67)	
Smokers	2 (33)	0 (0)	
Non-smokers	1 (17)	2 (33)	
Drug allergies, *n (%)*	4 (67)	3 (50)	1.000
Current serious SARS-CoV-2 infection or COVID-19, *n (%)*	5 (83)	3 (50)	0.545
Previous SARS-CoV-2 infection or COVID-19, *n (%)*	3 (50)	3 (50)	1.000
Renal insufficiency, *n (%)*	5 (83)	2 (33)	0.242
Transplant*, *n (%)*	3 (50)	2 (33)	1.000

*The data presented here have been cross-verified with hospital records; however, inconsistencies remain with some self-reported information. A resolution for these discrepancies is pending*

* Transplant includes all types of transplantations, covering both solid organ transplants and liquid transplants such as hematopoietic stem cell transplants and other cell-based therapies

In our analysis of renal insufficiency and transplant history, the patient’s auto-report was considered; however, when the patient answered: “I don’t know” (only one case of renal insufficiency), the EHR was considered. Considering the renal insufficiency, in 2 of the 12 cases there was disagreement between the patient’s self-report and the EHR: patients reported no renal insufficiency, but the EHR indicated that there was. Considering the transplant history, there was disagreement between the patient’s self-report and EHR in 4 of the 12 cases: in three of them, the patient stated that he had no history of transplantation, but the EHR indicated that there was, and in one case, the patient claimed to have transplant history, and the EHR did not confirm it.

Lastly, medication adherence was high, with no cases reporting forgotten doses or medication discontinuation. Only three cases reported carelessness with the timing of doses. Among those without possible or probable AE, two cases (33%) had some lapses in timing, while in the group with possible or probable AE, one case (17%) reported a lapse in the timing of medication administration (p = 0,505).

## Discussion

In this prospective cohort with COVID-19 outpatients who were at high risk of developing serious disease, initiation of molnupiravir was associated with significantly lower risks of all-cause mortality and disease progression, while demonstrating a good safety profile. To our knowledge, this is the first real-world study in Portugal and one of the few worldwide to monitor these outcomes through a prospective design.

Based on limited data regarding the safety and efficacy of oral antivirals for COVID-19, current guidelines prioritise their use for patients at high-risk of disease progression who do not require supplemental oxygen [24, 25]. Our study cohort reflects this prescription pattern in a real-world clinical practice scenario, and provided evidence supporting the use of molnupiravir in individuals at risk of severe disease - specifically, in those with severe immunodepression regardless of vaccination status, or at risk due to lack of COVID-19 infection or vaccination in the previous six months. Taking into account the outcome mortality, our findings suggested a positive effectiveness profile for molnupiravir, with no hospitalisations and/or deaths reported among treated patients up to the 29^th^ day after treatment initiation. Although this was the timeframe for assessing the effectiveness outcome, all patients were alive and could be contacted for the final safety follow-up at three months. This is consistent with the low incidence rates of hospitalisation and/or death observed in larger clinical trials such as the MOVe-OUT, which reported a 7.3% incidence rate in its interim analysis^2^ and a final reported incidence of 6.8% [11], reflecting reductions in hospitalisation and/or death risks by 50% and 30%, respectively, compared to placebo. Also, the PANORAMIC trial [26] reported an incidence rate of 1% in the molnupiravir-treated group, which is lower than those seen in the MOVe-OUT trial [11] but closer to the results of our study. Additionally, the PANORAMIC trial demonstrated minimal risk reduction in hospitalisation and death compared to placebo (adjusted OR 1.06 [95% BCI 0.81–1.41]). Altogether, those studies and ours indicate molnupiravir’s potential to reduce severe COVID-19 complications when administered early in the disease course and suggest that effectiveness may vary based on patient demographics and vaccination status (our study focused on vaccinated individuals, unlike the MOVe-OUT trial, which involved unvaccinated participants).

The effectiveness of molnupiravir in treating COVID-19 is further supported by real-world evidence indicating low hospitalisation and mortality rates. The real-world data from Evans et al. [19] reported a 4.1% incidence of severe outcomes in the treatment group, noting a 35% reduction in risk compared to untreated patients. Similarly, Paraskevis et al. [27] found that molnupiravir led to a hospitalisation rate of 5.1% and a notably low COVID-19-associated mortality rate of 1.2%, reinforcing the drug’s potential benefits The retrospective observational study by Wong et al. [15] also highlighted the protective effects of molnupiravir, reporting a significant reduction in all-cause mortality (HR 0.76 [95% CI 0.61–0.95]) and a consistent finding for hospitalisation rates (HR 0.98 [95% CI 0.89–1.06]). Focusing solely on the treated cohort, they reported mortality and hospitalisation incidences of 1.84% and 11.08%, respectively. Additionally, Park et al. [28] observed an even lower hospitalisation incidence of 0.8% with no deaths, while Czarnecka et al. [29] reported higher rates of hospitalisation and mortality at 14% and 2.8%, respectively, in a study where most participants were solid organ transplant recipients, highlighting variability in molnupiravir’s impact across different patient populations.

This evidence illustrates molnupiravir’s potential effectiveness in real-world settings, particularly among patients who might not be eligible for first-line antiviral treatments. Patients receiving molnupiravir often do so due to contraindications or potential adverse interactions with drugs like nirmatrelvir-ritonavir [30], particularly those on chronic medications such as warfarin or cyclosporine. Moreover, molnupiravir’s use in indiv iduals with renal insufficiency showcases its utility across a spectrum of patients with varying clinical profiles [31]. These factors underline the importance of molnupiravir as a versatile second-line therapeutic option for those at high risk of COVID-19 progression. The synergy between molnupiravir and widespread vaccination efforts likely also contributes to the reduced severity of disease outcomes seen in these studies. The altered immune response due to prior vaccination may enhance the therapeutic effects of molnupiravir, suggesting that when combined with preventive measures, the drug can effectively mitigate the impact of COVID-19 across diverse patient populations [17, 26]. Notably, when interpreting our results, we should also consider potential differences in baseline patient characteristics. Molnupiravir users were generally older (over 60), and half had experienced a previous SARS-CoV-2 infection. Our study adds to the literature on real-world effectiveness in specific contexts considering different variants of concern and population immune settings.

In our study, molnupiravir demonstrated a favourable safety and tolerability profile among patients with at least one risk factor for severe COVID-19. We identified a 50% incidence of AE attributed to molnupiravir. Comparatively, Tiseo et al. [32], Mazzitelli et al. [33], and Mutoh et al. [34] reported lower incidences of 21.1%, 6.9%, and 2.7%, respectively, including vaccinated patients, albeit with larger sample sizes than our study. In the MOVe-OUT trial [11], foundational to molnupiravir’s regulatory approval and conducted exclusively with unvaccinated patients, 30.4% reported at least one AE, with only 8.0% attributed directly to the drug. The most reported AE were diarrhoea, nausea, and dizziness, aligning with our findings, except for diarrhoea, which was notably absent in our study except in one case associated with antibiotic use.

Our study also highlighted nausea as the most frequently reported symptom, affecting 25% of patients, with 33% of these cases classified as serious. Other studies have frequently reported nausea but at lower incidences, ranging between 0.4% and 10.4% [11, 28, 29, 34]. The absence of diarrhoea in our study, associated with molnupiravir use, contrasts with its initial reporting of 1.7% in the MOVe-OUT trial [11] and a very low rate of 0.4% observed by Mutoh et al. [34]. Our findings of one patient reporting dysphagia and another stomach pain, the latter without recovery at the end of the three-month monitoring, resonate with Czarnecka et al.’s [29] 4.7% incidence of stomach pain. Vomiting was reported as a serious AE in only one patient, similar to findings from Gentil et al. [35]. Dizziness occurred in 17% of our patients, significantly higher than the 0.7% [35] and 2.9% [28] reported in previous studies. This heightened incidence may reflect variations in patient sensitivity or reporting practices across studies. Additionally, 17% of patients reported a bitter taste, recognised in the medical literature as dysgeusia - a term for taste alterations not listed in the SmPC. This could be linked to molnupiravir’s interaction with taste receptors, similar to how nirmatrelvir/ritonavir has been shown to activate a specific bitter taste receptor in the mouth [36]. Incidences in other studies range from 0.8% to 2.8% [28, 32, 35], which largely fall within the confidence interval of our study, confirming consistency with existing literature. Headache was reported by 17% of our patients, a rate that is 10 to 16 times higher than what was observed in previous studies [32, 35]. Additionally, the lack of skin and subcutaneous tissue disorders such as rash and urticaria in our study, despite their occasional mention in the SmPC and reports of incidences ranging from 0.4% to 2.8% in other research, highlights a discrepancy in AE profiles. Among the adverse events considered possibly or probably related to molnupiravir, nine were not listed in the SmPC, including symptoms such as dysphagia, nasal stuffiness, tiredness, and muscular pain. While most of these events were non-serious and self-limiting, their emergence reinforces the importance of post-marketing surveillance in detecting potentially unexpected safety signals. This highlights the added value of real-world data in identifying a broader range of possible adverse events that may not be captured in pre-authorisation trials. The COVID-19 rebound phenomenon noted with molnupiravir, with incidences from 1.8% to 7% [18, 32, 37], similar to those seen with nirmatrelvir-ritonavir, indicates a pattern of temporary viral suppression rather than outright eradication by these treatments. Lastly, Tiseo et al. [32] reported insomnia, leucopenia, and AST/ALT increase (two times upper limits of normal ULN) in just one patient each, highlighting the varied AE profile associated with molnupiravir.

Park et al. [28] reported that almost all AEs were of mild severity, and 4.2% were moderate. Despite this, they noted a 3.3% discontinuation rate due to AE. In contrast, our study recorded no discontinuation, highlighting the good tolerability of the antiviral used. Most AE with causality attributed to molnupiravir coincided with the prescribed 5-day treatment period, although some patients reported AEs that persisted beyond the close of monitoring (3 months). These enduring AE might reflect underlying symptoms of COVID-19 and other illnesses, potentially exacerbated after taking the medication, indicating that while some AE are attributed to molnupiravir, they could also represent the disease’s natural progression or exacerbation. Moreover, AE with causality was more frequently described in older individuals and those overweight, suggesting these factors might contribute, albeit to an unknown extent, to the occurrence of AE with molnupiravir. The lack of statistical significance in these findings is likely due to the small sample size of the study, which could mask true effects. However, the clinical significance of these observations should not be underestimated. AE with causality were more frequently described in older individuals. Concerning overweight, although there was no statistical difference, the clinical significance is noteworthy, as physiological changes in older age and altered drug metabolism in overweight individuals can influence the pharmacodynamics and pharmacokinetics of molnupiravir [9, 38].

Unlike other studies where treatment discontinuation affected outcomes, high adherence to molnupiravir treatment in our study underscores the acceptance of molnupiravir in a real-world setting and might have contributed to the observed effectiveness.

### Study strengths and weaknesses

Our study has several strengths. Firstly, the methodological approach combines patient self-reports with EHR, significantly enhancing the quality of data and their outcomes. Secondly, we implemented frequent monitoring intervals based on existing literature, specifically timed to capture both safety and effectiveness outcomes, ensuring systematic documentation and analysis of all relevant clinical data. This was critical in establishing a robust temporal relationship between molnupiravir exposure and the observed clinical outcomes. Thirdly, the duration of follow-up in our study extended to three months, a period three times longer than that considered in initial clinical trials, providing a more comprehensive assessment of the treatment’s medium-term safety. Fourthly, the study’s prospective design, specifically tailored to this medication, not only underscores its methodological robustness but also overcomes the common limitations of retrospective studies, as evidenced by the ENCePP’s recognition with the awarding of the study seal. Lastly, an expert clinical team performed the causality assessment of all AE, enabling a clearer understanding of the potential causal relationship between exposure and the development of AE.

Nevertheless, some limitations of our study should also be acknowledged. Firstly, the observational design, while comprehensive, cannot fully exclude the possibility of selection bias or confounding by indication, despite efforts to ensure that our cohort accurately represented the target patient population. Secondly, the possibility of recall bias cannot be ignored, although we attempted to minimise this through our structured follow-up strategies. Thirdly, being a single-cohort study without a control group limits our ability to draw definitive causal inferences from the observed outcomes. However, we sought to mitigate this limitation by discussing our findings against data from prior studies, thereby providing context and enhancing the interpretability of the observed outcomes. Fourthly, the clinical profile of our participants, who are at risk of progressing to severe COVID-19, may differ from those in larger trials, potentially affecting the generalisability of our results. For example, while major trials identified obesity as a common risk factor, our study highlighted older age as a more predominant risk factor, although obesity was also present. Lastly, being a second-line treatment option, which consequently led to fewer patients being recruited, could initially appear to skew the applicability of our findings. However, this can also be viewed as reflective of a real-world scenario where this medication is targeted at a specific subset of patients. Consequently, while the small sample size limited the statistical power of our analyses, reducing the precision of the study and potentially masking clinically significant effects, it does provide targeted insights relevant to those patients who would be eligible for this treatment.

The findings from our study underscore the critical need for active pharmacovigilance programs to monitor the long-term safety of oral antivirals, such as molnupiravir, which was withdrawn from the market after the completion of our study. Despite its withdrawal, the insights gained from examining its real-world use are invaluable. They not only inform potential future applications of molnupiravir in other clinical conditions but also enhance our understanding of its safety profile, which is essential for managing patients previously treated with the drug. These efforts contribute to a broader knowledge base, ensuring that past experiences with molnupiravir remain relevant and informative for ongoing medical research and future therapeutic strategies.

## Conclusion

In conclusion, our study appears to show that molnupiravir is safe and effective and may constitute an alternative for high-risk COVID-19 outpatients ineligible for first-line therapy. It was associated with a lower incidence of significant outcomes like all-cause mortality and/or hospitalisation among high-risk COVID-19 patients. Despite molnupiravir’s market withdrawal, understanding its long-term effects remains critical, as this knowledge could inform future clinical applications and research, potentially repurposing the drug for other viral infections.

## Electronic supplementary material

Below is the link to the electronic supplementary material.

Supplementary Material 1

## Data Availability

No datasets were generated or analysed during the current study.

## Abbreviations

AE: Adverse Event
BMI: Body Mass Index
CI: Confidence Interval
COVID-19: Coronavirus Disease 2019
DGS: Direção-Geral da Saúde (Portuguese Directorate-General of Health)
HER: Electronic Health Record
EMA: European Medicines Agency
ENCePP: European Network of Centres for Pharmacoepidemiology and Pharmacovigilance
ESOA-19: Estudo de Segurança de Antivirais em COVID-19
FDA: Food and Drug Administration
IQR: Interquartile Range
MedDRA: Medical Dictionary for Regulatory Activities
MOVe-OUT: Molnupiravir Phase 3 Study (official name of the pivotal trial)
NCT: National Clinical Trial (registry number)
RdRp: RNA-dependent RNA polymerase
RWE: Real-World Evidence
SARS-CoV-2: Severe Acute Respiratory Syndrome Coronavirus 2
SmPC: Summary of Product Characteristics
SPSS: Statistical Package for the Social Sciences
STROBE: Strengthening the Reporting of Observational Studies in Epidemiology
ULN: Upper Limit of Normal
WHO: World Health Organization
WHO-UMC: World Health Organization–Uppsala Monitoring Centre
